# An Experimental Study of 3D Electrode-Facilitated Particle Traffic Flow-Focusing Driven by Induced-Charge Electroosmosis

**DOI:** 10.3390/mi10020135

**Published:** 2019-02-18

**Authors:** Tianyi Jiang, Ye Tao, Hongyuan Jiang, Weiyu Liu, Yansu Hu, Dewei Tang

**Affiliations:** 1 State Key Laboratory of Robotics and System, Harbin Institute of Technology, Harbin 150001, China; jty_hit@hit.edu.cn (T.J.); jhy_hit@hit.edu.cn (H.J.); dwtang@hit.edu.cn (D.T.); 2 School of Electronics and Control Engineering, Chang’an University, Xi’an 710064, China; huyansu@chd.edu.cn

**Keywords:** microfluidic particle concentrator, continuous and switchable particle flow-focusing, composite electrode arrangement, induced-charge electroosmosis, field-effect flow control

## Abstract

In this paper we present a novel microfluidic approach for continuous, rapid and switchable particle concentration, using induced-charge electroosmosis (ICEO) in 3D electrode layouts. Field-effect control on non-linear electroosmosis in the transverse direction greatly facilitates a selective concentration of biological yeast cells from a straight main microchannel into one of the three downstream branch channels in our microfluidic device. For the geometry configuration of 3D driving electrode plates on sidewalls and a 2D planar gate electrode strip on the channel bottom surface, we briefly describe the underlying physics of an ICEO-based particle flow-focusing method, and provide relevant simulation results to show how gate voltage amplitude can be used to guide the motion trajectory of the concentrated particle stream. With a relatively simple geometrical configuration, the proposed microfluidic device provides new possibilities to controllably concentrate micro/nanoparticles in continuous flow by using ICEO, and is suitable for a high-throughput front-end cell concentrator interfacing with various downstream biosensors.

## 1. Introduction

In a number of biomedical and analytical applications, it is a crucial step to purify or extract target micro- or nano- particles from various sample matrices. To process samples with a low number of particles, a concentration step is usually imperative to abate the sample volume to an effective range (nL to μL) that microfluidic devices can handle [1,2]. Numerous methods have been developed concerning microfluidic particle concentration and manipulation in lab-on-a-chip systems such as direct current (DC) electrokinetics [3,4], alternating current (AC) electrohydrodynamics [5,6,7,8,9,10,11,12,13,14,15,16,17,18], dielectrophoresis [19,20,21,22,23,24,25], hydrodynamics, ultrasonic wave [26,27,28] magnetism, and ion concentration polarization [29,30,31].

Induced-charge electrokinetic phenomenon occurs as an applied electric field E induces a bipolar diffuse screening cloud adjacent to a polarizable solid surface immersed in electrolyte, then forces that induced double-layer (IDL) into an induced-charge electroosmotic (ICEO) flow [32,33,34,35,36,37,38,39,40,41,42,43,44,45]. This promising technique has been utilized to realize many microfluidic applications, including liquid pumping [33,46,47,48,49], mixing [50,51,52], as well as induced-charge electrophoresis of polarizable particles [53,54,55,56,57,58,59]. However, the use of ICEO fluid motion to achieve flexible particle manipulation has rarely been exploited. Recently, we reported position-controllable trapping of microscale particles on a bipolar metal strip in static flow condition by exploiting a tunable ICEO technique, termed the AC-flow field effect transistor (AC-FFET) [60]. To generate AC-FFET, a gate electrode (GE) is placed inside an externally-applied AC electric field for introducing biased ICEO slipping flow on its ideally polarizable surface, and can be flexibly addressed. By contrast with traditional methods, one important feature of AC-FFET is that a change in the gate voltage amplitude enables a variation in lateral ICEO flow profile above GE, greatly facilitating a flexible particle trapping technique [60]. 

**Figure 1:**
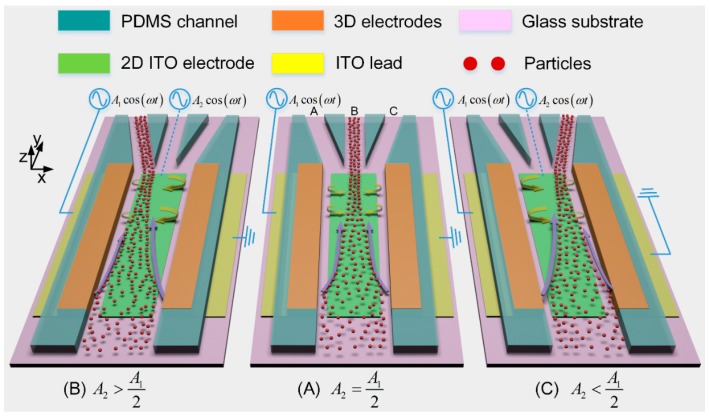
A 3D schematic of the presented microfluidic particle concentrator that is capable of continuously focusing the incoming particle samples into a switchable downstream branch channel by adjusting the voltage amplitude of the indium tin oxide (ITO) gate electrode (GE): (**A**) when the middle ITO electrode floats in potential, i.e., $A_{2}=\frac{A_{1}}{2}$, the two opposite induced-charge electroosmotic (ICEO) eddies above the electrode surface divert the trajectories of the incoming particles to the middle branch B; (**B**) when the biased gate voltage of ITO GE is more than its floating potential, i.e., $A_{2}>\frac{A_{1}}{2}$, the single dominating ICEO micro-vortex which is clockwise rotating above the electrode surface makes particles move into the left branch A; (**C**) when $A_{2}<\frac{A_{1}}{2}$, particles move into the right branch C due to the action of the counterclockwise rotating ICEO vortex.

Although AC-FFET is flexible in particle manipulation, in most situations, a microfluidic device that is able to focus incoming particle samples in a continuous pressure-driven fluid flow rather than a static condition is often of greater scientific significance [20,29,61]. Inspired by this, on the basis of AC-FFET, we present herein a novel microfluidic approach for continuous, rapid and switchable particle concentrating with 3D electrode layouts. The microfluidic device with composite electrode structure consists of a pair of face-to-face 3D silver-polydimethylsiloxane (Ag-PDMS) [62] driving electrodes fabricated on both sidewalls of the device channel and a planar indium tin oxide (ITO) metal strip acting as the gate electrode (GE), all of which are disposed along the longitudinal direction of the main channel bifurcating into three downstream branch channels (Figure 1).

The performance of the concentration device is validated by flow-focusing yeast cells of 5 μm in diameter suspended in low-conductivity KCl aqueous solution. By controlling the gate voltage amplitude, transverse ICEO convective rolls of adjustable flow profiles are induced above the surface of the planar GE, so that the device can continuously concentrate the incoming yeast cells into a switchable downstream branch channel (Figure 1). The proposed approach of continuous label-free sample processing provides a robust front-end concentration interface for a variety of biosensors and detection systems, without major limitation in downstream integration.

## 2. Mechanism of Switchable Particle Concentration by Using Alternating Current-Flow Field Effect Transistor (AC-FFET)

A bi-layer asymptotic model of ICEO convective flow has been proposed in our previous work [60], where a non-linear slip profile above the reciprocal resistor-capacitor (RC) time scale explains the stable and position-controllable trapping of yeast cells on the surface of a bipolar metal strip. In this study, by combining this non-linear ICEO slip profile with an incoming laminar flowing stream from the inlet to the outlets, we develop a novel microfluidic device to focus particle samples continuously from the suspending medium into a selective downstream branch channel (Figure 1). 

**Figure 2:**
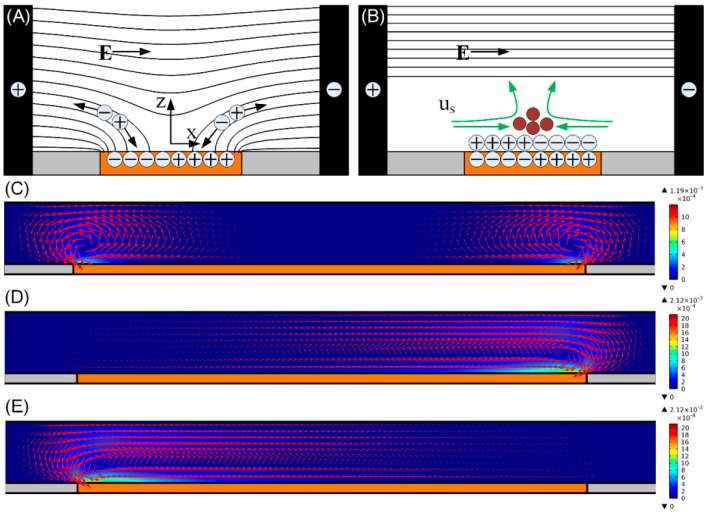
(**A**,**B**) Formation of ICEO around an ideally polarizable ITO metal strip, a floating ITO electrode (orange) is polarized by ionic current in a suddenly applied background electric field supplied by the sidewall 3D silver-polydimethylsiloxane (Ag-PDMS) driving electrodes (black), with the black lines and green arrows representing the electric field lines and ICEO flow velocity vectors, respectively. (**A**) Mobile counter-ions follow the electric field lines to the electrode surface; (**B**) at steady state, an induced double-layer (IDL) of dipolar nature and finite Debye length is formed at the metal/electrolyte interface, leaving only tangential bulk electric field forcing the IDL into ICEO convective flow. (**C**–**E**) A surface and arrow plot of ICEO flow field in the *x*-*z* plane for switchable particle concentrating (unit: m/s): (**C**) when the ITO electrode floats in potential, i.e., $A_{2}=A_{1}/2$, at *f* = 300 Hz and *A*_1_ = 30 V for concentrating particles into the middle branch; When different biased gate voltage *A*_2_ is imposed on the ITO GE at *f* = 30 Hz and *A*_1_ = 15 V; (**D**) *A*_2_ = 14.875 V for concentrating particles into the left branch; and (**E**) *A*_2_ = 0.125 V for concentrating particles into the right branch.

We first deal with the standard physical process of ICEO at the liquid/floating electrode interface. As Figure 2A shows, on switching the AC voltage wave on, initial normal component of electric field vector on the ITO electrode surface brings mobile counter-ions to the metal/electrolyte interface, where they pile up and form a dipolar IDL after a characteristic RC time scale $\tau_{RC}=aC_{D}/\sigma_{f}\left(1+\delta \right)=\varepsilon_{f}a/\sigma_{f}\lambda_{D}\left(1+\delta \right)$ (a=0.5 L) is the macroscopic length scale, *L* = 500 μm the width of ITO strip electrode, $C_{D}=\varepsilon_{f}/\lambda_{D}$ the diffuse layer capacitance, δ is the surface capacitance ratio) due to a force equilibrium between electrostatic attraction and thermal diffusion as shown in Figure 2B. The Debye length of IDL $\lambda_{D}=\sqrt{D\varepsilon_{f}/\sigma_{f}}$ lies in the range of 0.5–100 nm for aqueous solutions, where $D=2\times 10^{-9}\;m^{2}/s$ is the ionic diffusivity, while $\varepsilon_{f}=7.08\times 10^{-10}\;F/m$ and $\sigma_{f}=0.001\;S/m$ are the dielectric permittivity and electrical conductivity of the bulk fluid, respectively.

At steady state, an inhomogeneous distribution of the induced surface charge in the diffuse screening cloud fully repels the bulk field lines, so the ideally polarizable metal surface of the floating electrode behaves as an insulator from the perspective of an observer at very low frequencies (Figure 2B). The tangential field component acting on its own induced ionic charge in the IDL gives rise to two opposite ICEO eddies in the transverse *x*-*z* plane, resulting in a lateral flow stagnation line at the center of the electrode surface which can be exploited for particle concentration at proper conditions (Figure 2B). When the field frequency exceeds the surface-averaged reciprocal RC charging time $f_{RC-average}=\sigma_{f}\left(1+\delta \right)/2\pi C_{d}\left(0.5L\right)=37\;\mathrm{Hz}$ for the equivalent circuit of the interfacial double-layer capacitance coupled to the bulk resistance, incomplete double-layer charging takes place due to the relaxation process. As a consequence, the metal surface recovers to a perfect conductor for frequency beyond $f_{RC-average}$, and ICEO flow velocity decreases by half at $f_{RC-average}=37\;\mathrm{Hz}$.

Long–range ICEO micro-vortices constantly transport particles from the bulk fluid to the transversal flow stagnation region at the electrode center (Figure 2), where they can be stably trapped if the upward ICEO fluidic drag can be balanced by the downward buoyancy force [60]. In our previous work, we report that for field frequency above the reciprocal RC charging time $f_{RC-average}=37\;\mathrm{Hz}$, a spectrum of charging modes results in a nonlinear ICEO slip profile on the surface of the planar ITO electrode [60]. Since the nonlinear ICEO slip profile above $f_{RC-average}$ induces a negligibly small upward ICEO flow component, the downward buoyancy force is able to overcome the upward fluidic drag, which makes the cells entrained by bulk ICEO flow trapped at the center of the electrode surface.

Now we introduce the above idea of ICEO-based particle trapping into a continuous-flow microfluidic device, where an incoming Poiseuille stream of inlet flow velocity u_0_ is stably flowing from the inlet to the three downstream outlet branches (Figure 1). 

By energizing the sidewall 3D Ag-PDMS driving electrodes with an AC voltage signal $A_{1}\cos\left(\omega t\right)$ and keeping the potential of the planar ITO strip electrode $\frac{A_{1}}{2}\cos\left(\omega t\right)$ floating, ICEO flow is induced above the ITO electrode surface with a symmetrical flow profile perpendicular to that of the forward laminar stream carrying the particle sample to be concentrated. Here, f=2π/ω, ω and *A*_1_ are the field frequency, angular frequency, and voltage amplitude of the AC voltage, respectively.

From Figure 1A, as transported by transverse ICEO flow in the *x*-*z* plane from the bulk fluid to the electrode center, particles are at the same time dragged forward by the incoming Poiseuille flow from the inlet to the downstream outlets. A combined action of the transverse ICEO flow and the forward laminar flow diverts the trajectories of most incoming particles to branch B.

By adopting the tunable ICEO technique, AC-FFET [60], and energizing the ITO GE with a biased AC gate voltage $A_{2}\cos\left(\omega t\right)$ different from its floating potential $\frac{A_{1}}{2}\cos\left(\omega t\right)$, ICEO flow with an asymmetric flow profile is produced in the transverse direction. As a result of one dominating ICEO micro-vortex above the surface of the ITO electrode, the incoming particles move into the left (or right) branch as $A_{2}>\frac{A_{1}}{2}$ (or $A_{2}<\frac{A_{1}}{2}$) (Figure 1B,C).

As a consequence, by controlling the gate voltage amplitude and, therefore, generating transverse ICEO convection of adjustable flow profiles above the ideally polarizable surface, continuously focusing the incoming particle samples into a preferential branch channel is achieved in this microfluidic device (Figure 1).

To demonstrate the feasibility of the particle concentrator, numerical simulation of ICEO flow using Comsol Multiphysics 5.3a is performed in the *x*-*z* plane, with the detailed simulation procedure provided in Section 3.5. ICEO flow indeed yields vortices over the ITO electrode for achieving particle concentration in the transverse direction (Figure 2C–E). By comparing the cases with different biased gate voltage *A*_2_, broken symmetry of ICEO convective rolls occurs once the gate voltage *A*_2_ deviates enough from the natural floating potential of GE $\frac{A_{1}}{2}$.

When $A_{2}=\frac{A_{1}}{2}$, the two opposite eddies above the electrode surface (Figure 2C) can focus the incoming particles into the middle branch B. When $\frac{A_{1}}{2}<A_{2}<A_{1}$, the only clockwise ICEO vortex over the ITO diverts the trajectories of the incoming particles to the left branch A (Figure 2D). When $0<A_{2}<\frac{A_{1}}{2}$, particles move into the right branch C due to the action of the counterclockwise rotating ICEO vortex (Figure 2E).

## 3. Materials and Method

### 3.1. Device Geometry and Fabrication

**Figure 3:**
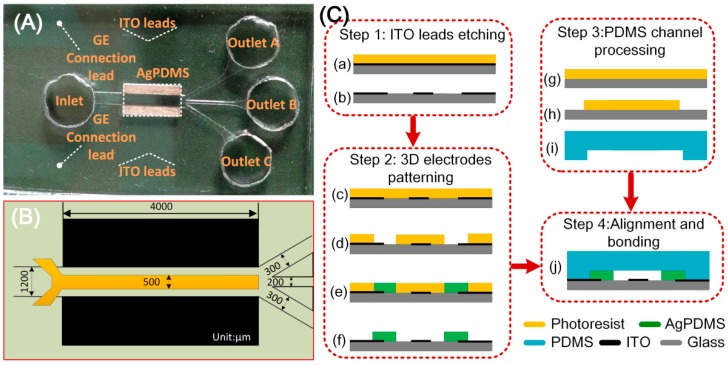
(**A**) A photograph of the microfluidic particle concentrator; (**B**) 2D illustration of the device geometry; (**C**) fabrication process of the experimental chip.

The microfluidic chip was composed of a main microchannel branching into three downstream outlets, as shown in Figure 3A. The four branch channels were connected to 4 respective reservoirs—the inlet, outlet A, outlet B and outlet C. The inlet and outlets were all of 6 mm in height and 6 mm in diameter, giving rise to volumetric capacity of about 170 μL. Under such capacity, the liquid level difference was able to drive a steady flow for about 15 min in the main channel. 

A pair of face-to-face 3D Ag-PDMS driving electrodes of 60 μm in thickness were fabricated on both sidewalls of the device channel, resulting in a 1.2 mm effective channel width (Figure 3B). The sidewall 3D electrodes were linked to the outer AC source through ITO leads. A planar ITO strip electrode of *L* = 500 μm in width and 200 nm in thickness was symmetrically disposed along the bottom centerline of the main channel and acted as GE. The length of Ag-PDMS electrodes was 4mm and equalled that of the ITO electrode. All the channels were 60 μm in height, while some other major dimensions of the configuration are shown in Figure 3B. 

The device was fabricated following the similar procedures as presented in our previous work [63]. In brief, the fabrication procedure consists of four steps as shown in Figure 3C: ITO leads etching, 3D electrodes patterning, PDMS channel processing, and alignment and bonding.

First, a clean ITO glass slide was laminated by negative dry film resist (Riston SD238, DuPont, Wilmington, DE, USA), followed by a photolithography process. The slide with patterned dry film was submerged into an etching solution to obtain the ITO leads, and then the dry film was stripped off by NaCO_3_ solution. After that, the slide was then laminated by two layers of dry film, followed by another photolithography process, hence generating the dry-film mold. Conductive Ag-PDMS gel [62] was then filled into the dry-film mold to form the sidewall 3D electrodes. After the fabrication of 3D Ag-PDMS electrodes, a PDMS microchannel was fabricated using conventional soft lithography method. Finally, the PDMS slab and the glass substrate with composite electrode structure were aligned under an optical microscope and bonded by oxygen plasma treatment.

### 3.2. Quantification of Particle Focusing Efficiency

Particle focusing efficiency $\eta_{X}$ for branch *X* among *A*, *B* and *C* can be defined as:

(1) $$\eta_{X}=\frac{N_{X}}{N_{total}}=\frac{N_{X}}{N_{A}+N_{B}+N_{C}}\times 100\%$$

Here $N_{total}=N_{A}+N_{B}+N_{C}$ is the total number of particles moving into the three branch channels as counted per half minute, and $N_{X}$ the number of particles moving into branch *X* over the same time period. 

### 3.3. Sample Preparation

The performance of the microfluidic particle concentrator was validated with yeast cells as the incoming particle samples, which were suspended in 0.001 S/m KCl aqueous solution. To prepare the sample solution of viable yeast cells, we suspended 50 mg of Baker’s dry yeast in 20 mL DI water to make a mixture and put this mixture in an oven at 30 °C for 1 h. After reactivation, we transferred 1 mL of the yeast suspension to a centrifuge tube. The yeast suspension was then washed and centrifuged with DI water for three times. After removing the supernatant, we transferred the precipitation of yeast cells to 1 mL of a 1 mS/m KCl aqueous solution. Prior to every experiment, the yeast cell solution was diluted 20 times in KCl solution of identical electrical conductivity, and a 5% bovine serum albumin (BSA) solution was applied to coat the microchannel for ~1 h to prevent the particles from adhering to any solid walls. 

### 3.4. Experimental Setup

A sample solution suspended with microparticles was first injected into the inlet. An AC signal, which was applied to the 3D electrodes through ITO leads, provided an AC electric field in the main channel. ICEO vortex flow was then formed on top of the ITO strip electrode, which dragged the particles from the surrounding medium to the ITO surface. 

The AC voltage signals applied to all the electrodes were produced by a series combination of a function generator (TGA12104, TTi, Buckinghamshire, UK) and a signal amplifier (Model2350, TEGAM, Geneva, OH, USA), and their waveforms were monitored by a digital oscilloscope (TDS2024, Tektronix, Beaverton, OR, USA).

A sinusoidal voltage signal $A_{1}\cos\left(\omega t\right)$ was applied to the left sidewall Ag-PDMS electrode, and the right one was grounded (Figure 1). The middle ITO GE was either floating in potential for concentrating particles into the middle branch B, or imposed with a biased voltage *A*_2_ cos(*ωt*) for selectively focusing particles into a desired side branch A or C.

**Figure 4:**
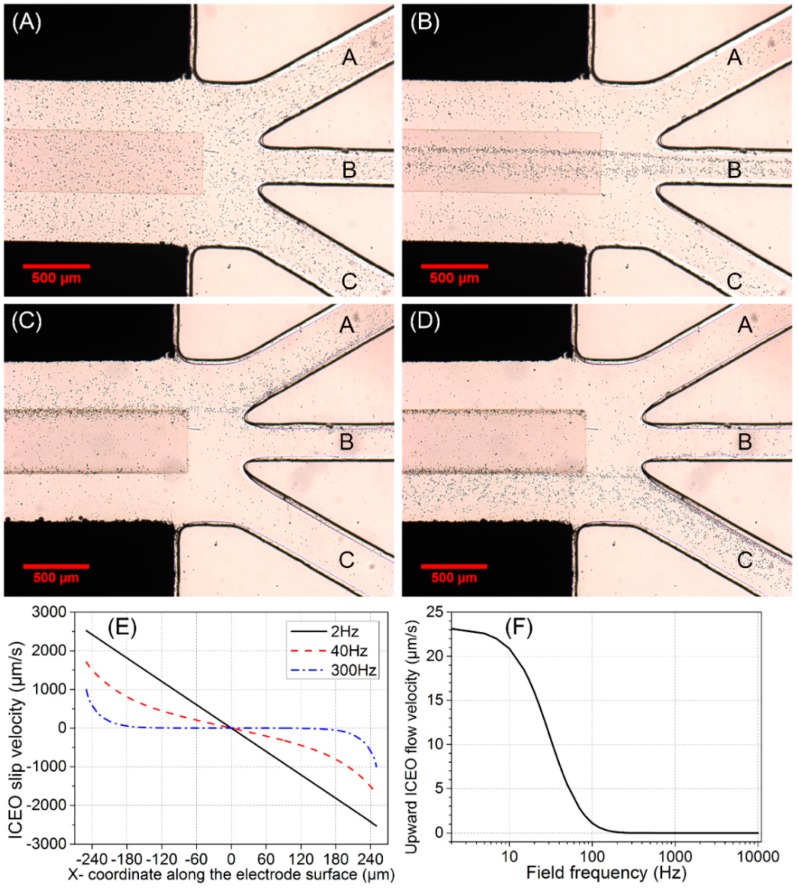
(**A**–**D**) Experimental observation of switchable particle concentrating at the main channel/branch channel junction for fixed inlet flow velocity *u*_0_ = 200 μm/s and different gate voltage *A*_2_: (**A**) initial distribution of yeast cells without AC power; (**B**) continuous-flow cell concentrating into the middle branch B by ICEO at *f* = 300 Hz and *A*_1_ = 25 V when the ITO electrode floats in potential, i.e., $A_{2}=\frac{A_{1}}{2}$; Continuous-flow cell concentrating into a desired side branch by AC-flow field effect transistor (AC-FFET) at *f* = 30 Hz and *A*_1_ = 15 V for different biased gate voltage *A*_2_, (**C**) for *A*_2_ = 14.875 V, 78% cells move into the left branch A, and (**D**) for *A*_2_ = 0.125 V, the trajectories of 78% cells are diverted to the right branch C. (**E**,**F**) Simulation results for *A*_1_ = 30 V: (**E**) ICEO slip profiles on the surface of ITO electrode at different field frequencies; (**F**) frequency-dependent upward ICEO flow velocity at *z* = 2.5 μm (half of the cell diameter) above the center of ITO electrode surface.

The yeast cell solution was injected through the microchannel with an approximate inflow velocity *u*_0_ = 200 μm/s at the channel inlet. Without a background AC electric field, the cells were distributed uniformly in the main channel and no obvious cell-concentrating phenomenon occurred (Figure 4A).

The behavior of particles was observed under an optical microscope (BX53, Olympus, Tokyo, Japan) and video-taped by using a Charge-coupled Device (CCD) camera (RETIGA2000R, Qimaging, Surrey, BC, Canada). As we concerned particle focusing near the outlet branches, the experimental observation window was chosen at the main channel/branch channel junction.

### 3.5. Simulation Procedure 

A standard bi-layer asymptotic model of the ICEO flow had been derived in our previous work, which made the electrokinetic problem readily decouple into one of electrochemical ion relaxation and another of viscous fluid flow originated by nonlinear electroosmotic slip at the polarizable surface. In this study, numerical simulation of the ICEO flow was acquired via Comsol Multiphysics 5.3a to enable a better understanding on how the particle concentrator works, with the simulation procedure provided below.

(a) Electrochemical ion relaxation

In the bulk, potential phasor $\tilde{\phi }$ satisfies the Laplace equation due to a constant conductivity throughout the entire channel:

(2) $$\nabla^{2}\tilde{\phi }=0$$

And $\tilde{E}=-\nabla \tilde{\phi }$ is the electric field phasor.

As for thin IDL approximation, the following RC boundary condition can be used to describe the double-layer charging process on the ideally polarizable metal surface of the floating electrode:

(3) $$\sigma_{f}\left(n\cdot \nabla \tilde{\phi }\right)=j\omega \frac{C_{D}}{1+\delta }\left(\tilde{\phi }-\tilde{V_{0}}\right)$$

Here $\delta =\frac{C_{D}}{C_{S}}$ is the surface capacitance ratio of the diffuse layer capacitance $C_{D}=\frac{\varepsilon_{f}}{\lambda_{D}}$ to the compact layer capacitance $C_{S}=0.2\;{F/m}^{2}$, ω=2πf the angular frequency of the applied sinusoidal voltage, $\tilde{V_{0}}=A_{1}/2$ the floating potential or $\tilde{V_{0}}=A_{2}$ the fixed potential of the middle ITO GE, ϕ the potential in the fluid bulk just outside the diffuse double-layer, $C_{0}=\frac{C_{D}}{1+\delta }$ the total area-specific capacitance at the metal/electrolyte interface, and n a unit normal vector on the surface of ITO electrode. 

Double-layer polarization around the driving electrodes was neglected due to a much lower characteristic charging frequency, so we imposed a fixed-potential boundary condition there:

(4a) $$\tilde{\phi }=A_{1}\;\left(\mathrm{On}\;\mathrm{the}\;\mathrm{left}\;\mathrm{electrode}\right)$$

(4b) $$\tilde{\phi }=0\;\left(\mathrm{On}\;\mathrm{the}\;\mathrm{right}\;\mathrm{electrode}\right)$$

The condition of zero normal electric current was imposed on insulating channel walls:

(5) $$n\cdot \nabla \tilde{\phi }=0$$

(b) Induced-charge electroosmotic flow

After solving for the electrostatic potential, the next step was to solve for the ICEO fluid flow velocity u satisfying the Stokes equation for creeping flow:

(6) $$\left\{ \begin{array}{l} -\nabla p+\nabla \cdot \left(\eta \left(\nabla u+{\left(\nabla u\right)}^{T}\right)\right)=0\\ \nabla \cdot u=0 \end{array}\right.$$

where *p* is the hydraulic pressure, and η=0.001 Pa⋅s the viscosity of KCL aqueous solution.

The expression for time-averaged ICEO slip velocity on the polarizable surface of the floating electrode can be derived from the generalization of Helmholtz–Smoluchowski formula

(7) 〈uslip〉=−εη〈ζEt〉=−εη12Re(ζ˜Et˜*)=12εfη11+δRe((ϕ˜−V0˜)(E˜−E˜⋅n⋅n)*)

Here < > means the time-averaged value in a harmonic field, * the complex conjugate operator, and Re() the real part of a complex number. ${\tilde{E}}_{t}=\left(\tilde{E}-\tilde{E}\cdot n\cdot n\right)$ is the tangential component of electric field vector on the electrode surface, and $\tilde{\zeta }=\frac{1}{1+\delta }\left(\tilde{V_{0}}-\tilde{\phi }\right)$ the induced zeta potential contributing to the ICEO fluid flow.

Besides, we applied no slip wall boundary condition on the surface of the driving electrodes and other insulating channel walls:

(8) u=0

(c) Modeling settings of the particle concentrator

To clarify the importance of ICEO flow effect on continuous and switchable particle concentrating, a numerical simulation employing COMSOL Multiphysics 5.3a was performed in the *x*-*z* plane.

The proposed 2D simulation model (Figure S1) was comprised of a microchamber with a pair of sidewall 3D Ag-PDMS driving electrodes. One planar indium tin oxid (ITO) electrode was deposited at the center of the channel bottom. The corresponding boundary conditions for electrostatics and hydrodynamics are summarized in Table S1, as shown in the Supplementary Information.

## 4. Results and Discussion

From Figure 4A,B, when ITO electrode floats in potential, yeast cells that stably move into middle branch B consist of two components: (1) incoming particles that are initially located above the surface of the ITO electrode; (2) particles that are not initially situated above the electrode surface but in close proximity to it can be transported by bulk ICEO vortical flows onto the electrode surface and subsequently enter branch B. However, incoming cell samples far away from the ITO electrode cannot be effectively influenced by ICEO flow, and hence move into the side branch A or C, which indicates the effective actuating range of ICEO convective flow is quite limited in this microfluidic device (Figure S2).

The simulated ICEO slip profile on the surface of ITO electrode transits gradually from a linear one in DC limit to a nonlinear one at 300 Hz beyond the RC relaxation frequency *f*_RC-average_ = 37 Hz (Figure 4E), resulting in negligibly small upward ICEO fluidic drag acting on the cell samples beyond 200 Hz (Figure 4F). 

We chose 300 Hz as an appropriate field frequency to concentrate cells into branch B by considering two aspects. On the one hand, below 200 Hz, the strong upward ICEO fluidic drag at the electrode center can overcome the downward buoyancy force, so cells make circulating motion with ICEO eddies. On another hand, at much higher frequencies, ICEO diminishes due to the mechanism of double-layer relaxation. Consequently, at 300 Hz, not only there is sufficient transverse ICEO driving force to push particles onto the electrode surface, but also the downward buoyancy force can overcome the upward ICEO fluidic drag to achieve stable cell trapping.

In contrast with the 25% particle-focusing efficiency in the absence of ICEO, η_B_ increases by 30% once AC background field of *f* = 300 Hz and *A*_1_ = 25 V is provided. With a further increase in *A*_1_ from 25 V to 30 V, however, η_B_ stays around 55%, partly due to the limited actuating range of ICEO eddies from vertical channel confinement (Figure S2A).

Besides the limited effective range of ICEO flow, another important characteristic of particle focusing is that cell samples cannot aggregate transversely to form a thin particle assembly line located at the center of the electrode surface, in stark contrast with our previous ICEO-based cell-trapping device [60]. What actually happens is that the incoming cells move along the longitudinal direction of the ITO electrode in the form of a wide particle stream instead of a thin assembly line (Figure 4B), due to the negative effect of vertical channel confinement on ICEO slip velocity along the ITO surface (Figure S3).

By employing the concept of AC-FFET and applying a second AC voltage signal *V*_2_(*t*) = *A*_2_ cos(*ωt*) to the middle ITO GE different from its floating potential *V*_2-0_(*t*) = *A*_1_/2 cos(*ωt*), we are able to continuously concentrate the incoming cells into the side branch at a low field frequency *f* = 30 Hz slightly below the double-layer relaxation frequency *f*_RC-average_ = 37 Hz (Figure 4C,D). Since a much lower frequency 30 Hz is chosen this time, the voltage amplitude *A*_1_ imposed on the sidewall Ag-PDMS electrode pair is lowered to 15 V for avoiding electrolysis and bubble formation. To induce effective AC-FFET phenomena, voltage amplitude *A*_2_ = 14.875 V or 0.125 V is applied to the ITO GE.

When *A*_2_ = 14.875 V is applied to GE, the trajectories of 78% cells are diverted to branch A (Figure 4C). Due to a voltage symmetry effect, when *A*_2_ = *A*_1_ − 14.875 V = 0.125 V is applied, 78% of cells move into branch C (Figure 4D).

From Figure 2D, when *A*_2_ = 14.875 V > *A*_1_/2, the field intensity in the right inter-electrode gap dominates over that in the left gap, which leads to a more intense double-layer charging effect on the right side of the electrode surface and, therefore, makes the right ICEO eddy dominate over the left one. Moreover, under the circumstance that *A*_2_ is sufficiently approaching *A*_1_, there is only a single dominating ICEO micro-vortex above the electrode surface (Figure 2D). This ICEO vortex is clockwise-rotating, acting as the role of the original right ICEO eddy (Figure 2C). The ICEO flow tends to push the cells from the right side of the ITO electrode to the left side, and has negligibly small upward flow component at the left side due to the vertical channel confinement effect (analysis not shown). Once particles are transported to the left side of the ITO electrode by transverse ICEO convection, they are pushed forward by the incoming Poiseuille stream with almost the same height due to the weak upward fluidic drag. Since they can never circulate back to the right side of the ITO electrode due to the negligible upward flow component at the left side, the cells finally move into branch A, which is in good accordance with Figure 4C.

Vice versa, from Figure 2E, under the condition of *A*_2_ = 0.125 V that is sufficiently approaching the grounding state, the counterclockwise ICEO eddy above the electrode surface diverts the trajectories of most cells to branch C, in qualitative agreement with Figure 4D.

Although we have made use of yeast cells of 5 μm in diameter suspended in low-conductivity KCl aqueous solution for confirming the actual device concentration performance in dynamic flow condition, our method is apt for dealing with any other micro/nanoscale sample as well. Particles having different mass density and geometric size may be collected at different height above the surface of GE, considering their positive effect on the gravitational force that acts downward. That is, particles of smaller radius or lower mass density would be arrested by the ICEO vortex at a larger vertical distance from the channel bottom surface, and thereby suffer from weakened lateral ICEO fluidic drag and enhanced forward transport compared to larger or heavier colloids. As a result, lighter or smaller incoming particle samples may form a wider colloid stream and transport more quickly along the channel length direction. As a consequence, a microfluidic separation device can be then developed by combining AC-FFET and distinct levels of ICEO arresting force in the lateral direction for continuously separating particle species maintained at different height away from the substrate surface, which serves potentially as an important topic of our future research.

## 5. Conclusions

In summary, we have demonstrated a microfluidic particle concentrator with composite 3D electrode structures, which utilizes AC-FFET to continuously focus the incoming particles into a switchable downstream branch channel at high throughput. When the ITO electrode floats in potential, transverse ICEO micro-vortexes with a symmetrical flow profile guide most of incoming yeast cells to move into the middle branch. By applying a second AC voltage to the ITO GE with amplitude deviating enough from its floating potential, the single ICEO eddy induced above the electrode surface successfully diverts the trajectories of incoming cells to the left or right branch at 30Hz, depending on whether the rotation direction of this dominating ICEO vortex is clockwise or counterclockwise. Such a continuous-flow and branch-switchable microfluidic particle concentrator presented here would be flexible for integration with various kinds of downstream devices, and is suited to front-end concentration interfaces for a variety of biosensors and detection systems.

## Supplementary Materials

The following are available online at http://www.mdpi.com/2072-666X/10/2/135/s1, Figure S1: 2D simulation model of ICEO flow (not to scale), Table S1: Boundary conditions used in numerical simulation, Figure S2: Influence of vertical channel confinement on the actuating range of ICEO flow: a surface and arrow plot of ICEO flow field in the *x*-*z* plane at *f* = 300 Hz and *A*_1_ = 30 V for different channel height *H* (unit: m/s), (A) *H* = 60 μm (experimental condition); (B) *H* = 500 μm (hypothetical); (C) *H* = 1000 μm (hypothetical), Figure S3: Simulation results at *f* = 300 Hz and *A*_1_ = 30 V: (A) ICEO slip profiles on the electrode surface for different channel height *H*; (B) ICEO slip velocity at *x* = 50 μm as a function of channel height, Video S1: Video clip of 3D electrode-induced field-effect-resettable particle flow-focusing.
