# Supplementary material for: An Experimental Study of 3D Electrode-Facilitated Particle Traffic Flow-Focusing Driven by Induced-Charge Electroosmosis
Source: Micromachines (Basel). 2019 Feb 18;10(2):135. doi: 10.3390/mi10020135 (PMC6412237; doi:10.3390/mi10020135)
Supplement: Supplementary file 1 [file micromachines-10-00135-s001.zip › supplementary.doc]

Supplementary Materials: An Experimental Study of 3D Electrode-Facilitated Particle Traffic Flow-Focusing Driven by Induced-Charge Electroosmosis

Tianyi Jiang ^1^, Ye Tao ^1,^*, Hongyuan Jiang ^1^, Weiyu Liu ^2,^*, Yansu Hu ^2^ and Dewei Tang ^1^

^1^	State Key Laboratory of Robotics and System, Harbin Institute of Technology, Harbin 150001, China; jty_hit@sina.com (T.J.); jhy_hit@sina.com (H.J.); dwtang@hit.edu.cn (D.T.)

^2^	School of Electronics and Control Engineering, Chang’an University, Xi’an 710064, China; huyansu@chd.edu.cn

*****	Correspondence: tarahit@gmail.com (Y.T.); liuweiyu@chd.edu.cn (W.L.);
Tel.: +86-0451-86-418-028 (Y.T.); +86-029-82-334-543 (W.L.)

1. Simulation Procedure

**Figure S1.** 2D simulation model of ICEO flow (not to scale).

**Table S1.** Boundary conditions used in numerical simulation.

| **Index number** | **Name** | **Electrical Boundary Condition for Equation (2)** | **Flow Boundary Condition for Equation (6)** |
| --- | --- | --- | --- |
| 1 | Left driving electrode | Equation (4a) | Equation (8) |
| 2 | Floating electrode | Equation (3) | Equation (7) |
| 3 | Right driving electrode | Equation (4b) | Equation (8) |
| 4 | Insulating wall | Equation (5) | Equation (8) |

2. Simulation Result

2.1. Influence of Vertical Channel Confinement on Actuating Range of ICEO Flow

In our previous work on ICEO based trapping of yeast cells above a bipolar metal strip, the effective actuating range of ICEO flow attains more than half of the electrode width on both sides of the electrode. However, the actuating range of ICEO eddies in this continuous-flow device is limited to great extent (Figure 4B), and the main reason accounting for this negative effect is the occurrence of vertical channel confinement (Figure S2).

Figure S2 shows the simulated ICEO flow field for different channel height. When the dimension of the channel height H matches with that of the electrode width L (Figure S2B,C), e.g., *H* = *L* = 500 μm, *H* = 2*L* = 1000 μm, ICEO can nearly affect every cell sample within the entire chamber. In the presence of severe vertical channel confinement (Figure S2A), e.g., *H* = 60 μm < *L*, however, the actuating range of ICEO flow is limited to a small region in the immediate vicinity of electrode edge, which is in qualitative agreement with Figure 4B. At an extremely low channel height *H* = 60 μm as the actual experimental condition, it is the viscous boundary layer at the insulating channel top wall that suppresses both the flow velocity and actuating range of ICEO convective rolls.

2.2. Influence of Vertical Channel Confinement on ICEO Slip Velocity

We even lower the inlet flow velocity to 50 μm/s, so there is about 80 s for the cells to move transversely from the electrode edge to the electrode center by ICEO. However, a similar wide particle stream (results not shown) is still obtained as in Figure 4B.

**Figure S2.** Influence of vertical channel confinement on the actuating range of ICEO flow: a surface and arrow plot of ICEO flow field in the *x*-*z* plane at *f* = 300 Hz and *A*_1_ = 30 V for different channel height H (unit: m/s), (**A**) *H* = 60 μm (experimental condition); (**B**) *H* = 500μm (hypothetical); (**C**) *H* = 1000μm (hypothetical).

**Figure S3.** Simulation results at *f* = 300 Hz and *A*_1_ = 30 V: (**A**) ICEO slip profiles on the electrode surface for different channel height *H*; (**B**) ICEO slip velocity at *x* = 50 μm as a function of channel height.

From Figure S3, the vertical channel confinement also has a negative impact on the ICEO slip velocity along the electrode surface. In the extreme case of very low channel height *H* = 60 μm, the magnitude of slip velocity decays to great extent as compared to other normal cases without vertical channel confinement (Figure S3A). The electrical insulation condition Equation (5) allows no normal electric current to penetrate into the channel top wall, while the RC charging conditon Equation (3) permits normal electric field component to emanate from the surface of ITO electrode above the reciprocal RC time scale. If the two boundaries including the channel top wall and the electrode surface are in close proximity to one another, the two paradoxical electric boundary conditions interfere with each other and therefore result in a decay in ICEO slip velocity (Figure S3A). Figure S3B presents the H-dependent slip velocity at a point 50μm away from the center of the electrode surface (*x* = 50 μm). For *H* = 60 um, slip velocity at *x* = 50 μm diminishes and is far less than 1μm/s, which indicates the cell samples can never enter the inner region of electrode surface by ICEO even if the inlet flow velocity is sufficiently slow, resulting in the observed wide particle stream moving into branch B (Figure 4B).

In DC limit, the RC boundary condition Equation (3) is reduced to Equation (5), so the electrical boundary conditions on the channel top wall and the ITO surface will no longer be in conflict at very low frequencies. Consequently, in this concentration device with severe vertical channel confinement, there is still strong ICEO flow on the electrode surface below the inverse RC time scale, which renders particle trapping unstable.

3. Mechanism of AC-FFET

AC-FFET is governed by different mechanisms in distinct frequency ranges as has been discussed in our previous work. In DC limit, electric field from GE is completely screened by ionic charge in the diffuse screen cloud and hence GE behaves as an insulator from the perspective of an observer, surface potential and electric field outside the IDL cannot redistribute due to the deviation of gate voltage from the floating potential. That is, in DC limit, imposing a gate voltage on the ITO electrode implies a direct control on the zeta potential across the IDL without a perturbation on the surrounding field distribution, and an analytical solution of ICEO slip velocity on the surface of GE in DC limit in a similar geometrical configuration has been derived in our previous work .

Below 30 Hz, however, it is very facile for bubble generation to take place, which causes great harm to the performance of this particle concentrator. For 30 Hz beyond the DC limit, the biased voltage *A*_2_ of GE penetrates into the outside of the Debye layer, which causes left-right asymmetry in the conduction current flowing into the IDL, forming another mechanism of AC-FFET.

It is hard to obtain an analytical solution once the field frequency is increased above DC limit, however, since the electric field from GE penetrates outside the IDL. Instead, we apply full numerical analysis under this circumstance, as shown in Figure 2D,E.

References

[1] Y. Ren, W. Liu, Y. Jia, Y. Tao, J. Shao, Y. Ding, et al., Induced-charge electroosmotic trapping of particles, Lab on a Chip, 15(2015) 2181-91.

© 2018 by the authors. Submitted for possible open access publication under the terms and conditions of the Creative Commons Attribution (CC BY) license (http://creativecommons.org/licenses/by/4.0/).
